# Nature's best vs. bruised: A veggie edibility evaluation database

**DOI:** 10.1016/j.dib.2025.111483

**Published:** 2025-03-19

**Authors:** Bidisha Samanta, Sriparna Banerjee, Ranadhir Das, Sheli Sinha Chaudhuri, Khalifa Djemal, Amir Ali Feiz

**Affiliations:** a Electronics and Telecommunication Engineering Department, Jadavpur University, Kolkata, India; b Paris-Saclay University, Université d'Évry, IBISC, Evry, 91020, France; c Paris-Saclay University, Université d'Évry, LMEE, Evry, 91020, France

**Keywords:** Automated vegetable edibility estimation, Database comprising vegetables having varied edibility, Computer vision

## Abstract

In the realm of evaluating vegetable freshness, automated methods that assess external morphology, texture, and colour have emerged as efficient and cost-effective tools. These methods play a crucial role in sorting high-quality vegetables for both export and local consumption, significantly impacting the revenue of the food industry worldwide. Researchers have recognized the importance of this area, leading to the development of various automated techniques, particularly leveraging advanced deep learning technologies to categorize vegetables into specific classes. However, the effectiveness of these methods heavily relies on the databases used for training and validation, posing a challenge due to the lack of suitable datasets.

Specifications Table

**Table:** 

Subject	Smart Agriculture, Vegetable freshness evaluation, Deep Neural Networks
Specific subject area	Automated Vegetable freshness quality evaluation
Type of data	Images of vegetables from Fresh to Damaged to Severely Damaged
Data acquisition	Images of vegetables included in this dataset are acquired using varied camera angles, non-uniform illumination and consistent white background.
Data format	Raw
Dataset preparation	Various freshness stages of vegetables were captured using the high-resolution rear cameras of three different mobile phones. These images were saved in JPG format after resizing to a fixed dimension of 256 × 256 using a Python script. The resized images were organized into six folders: Cowpea, Snake Gourd, Spiny Gourd, Ivy Gourd, Taro root and Turmeric. Within these folders, the vegetable images were further categorized into six subfolders based on their freshness level: Fresh, Damaged, and Severely Damaged. All images were captured under different lighting conditions against a white background. This meticulously curated vegetable image dataset serves a vital purpose in the testing, training, and validation of automated models designed for evaluation of freshness levels of vegetables. Researchers and developers can utilize this dataset to enhance the accuracy and efficiency of their vegetable-related projects, benefiting from the diverse quality and type variations captured in the images.
Data collection	Images of vegetables from Fresh to Damaged to Severely Damaged
Data source location	This dataset is prepared at Jadavpur University, Kolkata, India. 22.4989*°N, 88.3714°E*
Data accessibility	Repository name: Nature's Best vs. Bruised: A Veggie Evaluation Data identification number: 10.17632/b2mvj3kjfx.1 Direct URL to data: https://data.mendeley.com/datasets/b2mvj3kjfx/1
Related research article	None

## 1 Value of the Data

• This database contains a total of **4464** high-quality images of six different types of vegetables namely, Cowpea, Snake Gourd, Spiny Gourd, Ivy Gourd, Taro root and Turmeric.
• Images included within each vegetable category are further categorized into three classes namely, Fresh, Damaged, and Severely Damaged.
• The provided dataset offers a valuable resource for researchers working on deep learning solutions focused on classifying vegetables according to their freshness levels.
• The dataset holds immense potential in the development of high-quality vegetable freshness evaluation applications.

## 2 Background

The principle objective behind the curation of this database lies in the development of an automated vegetable edibility estimation method. In order to design any automated method, apart from the architecture of the method, another crucial parameter based on which the performance efficiency of the designed method is largely dependent on lies in the varsities in characteristics of samples used to train the method. As the absence of proper database often hinders the development of proper methods mostly in computer vision domain, so this database is specifically curated to mitigate this limitation.

## 3 Data Description

Trading of vegetables contributes in earning a significant revenue by the agricultural industry. Exporting vegetables often takes a lot of time which significantly impact the freshness of vegetables. Hence automatic sorting of vegetable in a non-invasive manner has become essential for cost effective and time efficient processing. Due to the overwhelming performance achieved by the advanced deep neural technologies in various automated tasks, nowadays researches have come up with automated designs to efficiently classify the edibility of vegetables be leveraging the advanced deep learning techniques [[1], [2], [3], [4], [5]].

The research has pioneered the creation of a unique database named ``Nature's Best vs. Bruised: A Veggie Evaluation,'' featuring **4464** images representing six commonly consumed vegetables in India: Turmeric (Scientific name: Curcuma longa), Spiny Gourd (Scientific name: Momordica dioica), Taro root (Scientific name: Colocasia esculenta), Ivy Gourd (Scientific name: Coccinia grandis), Snake Gourd (Scientific name: Trichosanthes cucumerina) and Cowpea (Scientific name: Vigna sinensis Savi).

These specific vegetables were chosen due to their widespread consumption and affordability, particularly in rural areas, and notably, they were absent from existing databases. These particular six types of vegetables are chosen because of the diversities in their growing characteristics which portray how the degradation nature of the shoot vegetables differ from that of the root vegetables. Out of the six types of vegetables, Taro root and Turmeric are root vegetables while the other vegetables grow above the ground. The sample images of this newly curated database are shown in table format containing all the stages from Fresh to Damaged to Severely Damaged where Tables 1, 2, 3, 4, Table 5, Table 6 holds Turmeric, Spiny Gourd, Taro root, Ivy Gourd, Snake Gourd, Cowpea images, respectively.

**Table 1:** Description of **Turmeric** sample images included in the nature's best vs. bruised: a veggie evaluation dataset.

**Table 2:** Description of **Spiny Gourd** sample images included in the nature's best vs. bruised: a veggie evaluation dataset.

**Table 3:** Description of **Taro Root** sample images included in the nature's best vs. bruised: a veggie evaluation dataset.

**Table 4:** Description of **Ivy Gourd** sample images included in the nature's best vs. bruised: a veggie evaluation dataset.

**Table 5:** Description of **Snake Gourd** sample images included in the nature's best vs. bruised: a veggie evaluation dataset.

**Table 6:** Description of **Cowpea** sample images included in the nature's best vs. bruised: a veggie evaluation dataset.

The sampling population of different types of vegetables considered for curating this database are:

1. Cowpea: 100 Samples
2. Ivy Gourd: 61 Samples
3. Snake Gourd: 99 Samples
4. Spiny Gourd: 104 Samples
5. Taro root: 48 Samples
6. Turmeric: 94 Samples

These are the number of samples whose images taken in different angles are included in the curated database. No additional augmentation is performed to increase the number of samples in the database. The inter-class variations in the number of samples makes the database imbalance which facilities its usage in evaluating the performances of the machine learning algorithms in handling imbalance dataset. However any user can perform augmentation and synthetically generate samples based on their requirements.

**Fig. 1:**
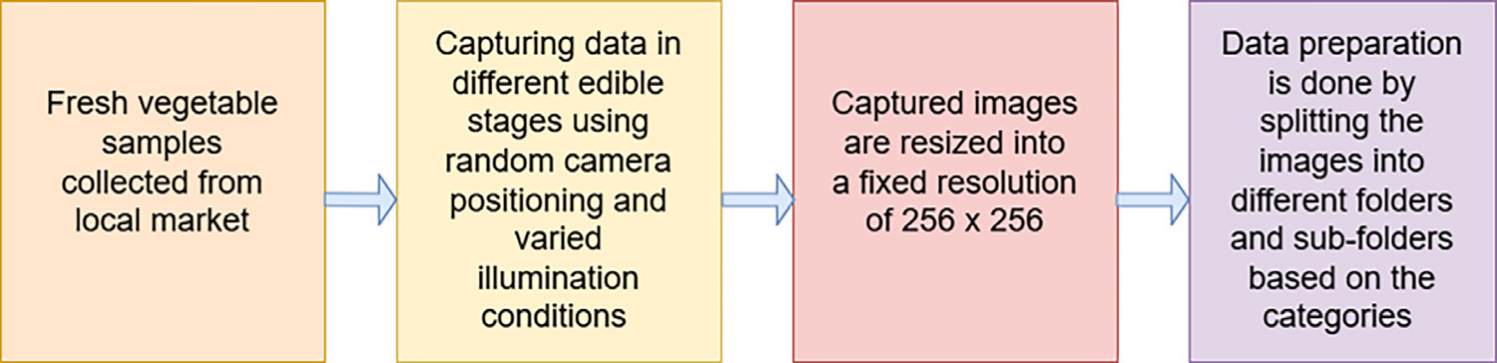
Steps adapted to curate the nature's best vs. bruised: a veggie evaluation dataset.

The images within our database authentically capture the natural progression of these vegetables, showcasing their transition from Fresh state to Damaged to Severely Damaged conditions, without any external manipulation. We classified these images into three distinct categories: Fresh, Damaged, and Severely Damaged. Fig. 1 displays the curation process of this database.

This comprehensive database stands as a valuable resource for researchers in the field, addressing the crucial need for robust datasets and facilitating advancements in automated vegetable quality assessment techniques.

Examples of images included in this newly curated database are given in the following Tables:

## 4 Experimental Design, Materials and Methods

The high-definition rear cameras of OnePlus7T, Redmi Note 9 pro max and OnePlus Nord mobile phones were employed to capture the photographs of the vegetables. After capturing the images, they are resized to a fixed dimension of 256 × 256 using a python script and are stored in appropriate folders in .jpeg format. Table 7 contains the consolidated device camera specifications which are utilized to capture images.

**Table 7:** Specification of image acquisition devices.

Sl.No	Properties	Camera 1	Camera 2	Camera 3
1	Device name	OnePlus7T	Redmi Note 9 pro max	OnePlus Nord
2	Camera specifications	**Rear camera** 48 megapixel (f/1.6, 0.8 micron) +16 megapixel (f/2.2) +12 megapixel (f/2.2) **Front camera** 16 megapixel (f/2.0, 1.0 micron)	**Quad camera** 64 megapixel (f/1.9, 0.8 micron) +8 megapixel (f/2.2) +5 megapixel (f/2.4) +2 megapixel (f/2.4)	**Quad camera** 48 megapixel (f/1.8, 0.8 micron) +5 megapixel (f/2.3) +5 megapixel (f/2.4) +2 megapixel (f/2.4)

### 4.1 Materials or Specification of Image Acquisition System

**OnePlus7T camera specifications**: 48 MP Ultra-wide Triple camera

**Redmi Note 9 pro max camera specifications:** AI Quad camera array 64 MP+8 MP+5MP+2MP

**OnePlus Nord camera specifications**: 48 OIS+8+5+2 MP Quad Camera, 32+8 MP Dual Front Camera

### 4.2 Method

All the vegetable species considered for designing this database are brought from local markets in fresh condition and are transformed from Fresh to Damaged to Severely Damaged stages under constant supervision in the laboratory. All of the steps adapted for the curation of this database is shown in Fig. 1 and Table 9 contains all the detailed information regarding the inter-class distribution among the various vegetable species considered while designing this database. The images included in this database are captured using varied camera angles, non-uniform illumination and consistent white background. The images captured for each class under each vegetable category are included into suitable folders after resizing them into a fixed resolution of 256 × 256 pixels and detailed information about all the image properties of this database is mentioned in Table 8. The inclusion of these diverse types of samples have enhanced the real-life effectiveness of this database enabling it to serve as a reliable one to perform training, validation and testing of methods designed to automatic freshness evaluation of vegetables.

**Table 8:** Images details and quality.

Sl.No.	Image details	Image quality
1	Dimension	256 × 256 pixels
2	Height	256
3	Width	256
4	Vertical resolution	72 dpi
5	Horizontal resolution	72 dpi
6	Bit depth	24
7	Resolution unit	2
8	Colour representation	sRGB

**Table 9:** Nature's best vs. bruised: a veggie evaluation: dataset details.

Classes	Vegetable Species	Camera angles, illumination and image background specifications	Number of images included under each class for each vegetable species
Fresh Damaged Severely Damaged	**Cowpea (***Scientific name: Vigna sinensis Savi)*, **Snake Gourd** (*Scientific name: Trichosanthes cucumerina)*, **Spiny Gourd** (*Scientific name: Momordica dioica)*, **Ivy Gourd** (*Scientific name: Coccinia grandis)*, **Taro root** (*Scientific name: Colocasia esculenta)* and **Turmeric** (*Scientific name: Curcuma longa)*	Images are captured using varied camera angles, non-uniform illumination conditions in the presence of artificial light and using consistent white background, thereby ensuring the diversity in samples.	**Fresh**
			*Cowpea:203*
			*Snake Gourd:122*
			*Spiny Gourd:418*
			*Ivy Gourd: 246*
			*Taro root:228*
			*Turmeric:350*
			**Damaged**
			*Cowpea: NIL*
			*Snake Gourd:124*
			*Spiny Gourd:487*
			*Ivy Gourd:254*
			*Taro root:186*
			*Turmeric:335*
			**Severely Damaged**
			*Cowpea:204*
			*Snake Gourd:123*
			*Spiny Gourd:420*
			*Ivy Gourd:254*
			*Taro root:186*
			*Turmeric:324*

## Limitations

None.

## Ethics Statement

The research presented herein does not involve human or animal subjects. All images utilized in this work were self-acquired and do not include any sourced from social media platforms. Additionally, no external funding was received for the completion of this research.

## Funding

This work is not funded by any sources.

## CRediT authorship contribution statement

**Bidisha Samanta:** Methodology, Data curation, Investigation. **Sriparna Banerjee:** Conceptualization, Methodology, Data curation, Investigation, Writing – review & editing, Validation. **Ranadhir Das:** Methodology, Data curation. **Sheli Sinha Chaudhuri:** Supervision. **Khalifa Djemal:** Supervision. **Amir Ali Feiz:** Supervision.

## Data Availability

Mendeley DataNature's Best vs. Bruised: A Veggie Evaluation (Original data).
